# Doing masculinity, not doing health? a qualitative study among dutch male employees about health beliefs and workplace physical activity

**DOI:** 10.1186/1471-2458-10-712

**Published:** 2010-11-19

**Authors:** Petra Verdonk, Hannes Seesing, Angelique de Rijk

**Affiliations:** 1 Institute for Public Health Genomics, Faculty of Health, Medicine and Life Sciences, School Caphri, Maastricht University, Universiteitssingel 5, 6229 ES Maasstricht, The Netherlands; 2 Department of Social Medicine, Faculty of Health, Medicine and Life Sciences, School of Public Health and Primary Care, School Caphri, Maastricht University, Universiteitssingel 40, Maastricht, The Netherlands; 3 Gezondheidscentrum Didam, Panhuis 52, Didam, The Netherlands

## Abstract

**Background:**

Being female is a strong predictor of health promoting behaviours. Workplaces show great potential for lifestyle interventions, but such interventions do not necessarily take the gendered background of lifestyle behaviours into account. A perspective analyzing how masculine gender norms affect health promoting behaviours is important. This study aims to explore men's health beliefs and attitudes towards health promotion; in particular, it explores workplace physical activity in relation to masculine ideals among male employees.

**Methods:**

In the Fall of 2008, we interviewed 13 white Dutch male employees aged 23-56 years. The men worked in a wide range of professions and occupational sectors and all interviewees had been offered a workplace physical activity program. Interviews lasted approximately one to one-and-a-half hour and addressed beliefs about health and lifestyle behaviours including workplace physical activity, as well as normative beliefs about masculinity. Thematic analysis was used to analyze the data.

**Results:**

Two normative themes were found: first, the ideal man is equated with being a winner and real men are prepared to compete, and second, real men are not whiners and ideally, not vulnerable. Workplace physical activity is associated with a particular type of masculinity - young, occupied with looks, and interested in muscle building. Masculine norms are related to challenging health while taking care of health is feminine and, hence, something to avoid. Workplace physical activity is not framed as a health measure, and not mentioned as of importance to the work role.

**Conclusions:**

Competitiveness and nonchalant attitudes towards health shape masculine ideals. In regards to workplace physical activity, some men resist what they perceive to be an emphasis on muscled looks, whereas for others it contributes to looking self-confident. In order to establish a greater reach among vulnerable employees such as ageing men, worksite health promotion programs including workplace physical activity may benefit from greater insight in the tensions between health behaviours and masculinity.

## Background

A large body of literature points towards sex differences in health and illness. These biological health outcomes occur in a pattern of gendered social interactions, namely expectations of how men and women should behave, and in practices that treat men and women of various ages, ethnic and social classes differently [1,2]. Research must therefore address both sex and gender [3,4]. In many countries, including the Netherlands, men's life expectancy is shorter than women's. Gender differences in lifestyle factors play an important role in leading causes of death in the Western world such as coronary heart disease (CHD) and cancer. Health promoting interventions need to address these differences in an equitable manner in order to be more successful. However, gender knowledge is insufficiently translated into interventions [5] and the gendered background of lifestyle behaviours, including participation in sports and exercise, is hardly taken into account. The aim of this study is to explore health beliefs and attitudes towards health promotion, in particular workplace physical activity, as well as how these beliefs are related to masculine ideals in a sample of Dutch male employees. Gender is one of the ways in which social practices are ordered [6] and certain conceptualizations of masculinities in some contexts are harmful to men's health whereas other conceptualizations may be beneficial [7-9].

### Gender, sports and aerobics/fitness

For a long time, competitive sports and exercise have been almost exclusively male activities. This has changed and currently, participation rates of Dutch men and women are rather similar (69% versus 72%) [10,11]. Sex differences do exist in the Netherlands regarding participation in and popularity of sport types. The three most popular sport types in 2007 and their participation rates - including incidentally - are: (1) swimming (32% of the men, 40% of the women); (2) cycling (men 27%, women 19%), and; (3) aerobics/working out (men 18%, women 26%). These three categories are also the three most popular types of sports and exercise among people of working age (20-64 years) [11]. Even though participation rates do not differ largely between men and women, reasons for participation may differ. Women prefer more often recreational types of sports while men dominate in team sports and competitive sports, and men engage more often than women in risky sports such as racing [11-13].

Workplaces have potential for lifestyle interventions because employees spend more than one-third of their waking hours, men more than women, at work. In the past decade in the Netherlands, employers have begun to invest in long-term programs to promote employees' health, well-being, and productivity [e.g. [14]]; an increasing number of organizations offer workplace physical activity to their employees. In the Netherlands, 2 million people out of approximately 18 million citizens participate in aerobics or work out in registered gyms and two out of three gyms offer workplace physical activity programs [10]. Literature regarding participation in workplace physical activity programs is scarce. A Dutch study indicates that between 8 and 22% of employees actually participate at least once per month in workplace physical activity programs: young employees (< 30 years) have higher participation rates; employees with a low income participate less often, and no gender differences in participation exist [15]. Men participate relatively less often than women in workplace physical activity programs compared to their labour market participation, but little is known about how such programs are experienced.

### Men's health behaviours

Men are often presented both *at risk *and as *risk-takers *[16], while being female is a strong predictor of health promoting behaviours [2,13,17,18]. Doyal states that maleness can be an advantage to health because men have more often privileged access to resources than women. But being male is also referred to as a 'mixed blessing' because in many cultures, men are required to take health risks [1]. The emergence of the 'male breadwinner' in the twentieth century led to more men in potentially life-threatening jobs [2,13,19,20]. Furthermore, men's choices to deal with their stress are often not health promoting [21]. Men engage more often than women in risk behaviours such as smoking, dangerous sexual activities, substance abuse, not using safety belts, and not getting health screenings [2,13,18,21]. Men's higher alcohol consumption rates contribute to their higher mortality rates at all ages and alcohol is often the cause of accidents or workplace injuries [22]. In a qualitative study among men conducted in the UK, conventional masculine ideals prevailed in men's perception of dietary messages: healthy eating is 'boring' and does not provide the energy for manly work [23]. Men are also less likely to seek help [24-26] or to perceive themselves at risk for health problems [13]. Not paying attention to and being silent about 'minor' or 'trivial' symptoms are a practice of masculinity [27]. However, this is not necessarily a disinterest in health. Men may closely self-monitor their symptoms and collect information, to decide whether they can 'fix' it themselves or need help in order to continue their regular activities [28].

Several explanations exist for gender differences in risk-taking behaviour [25]. First, sexual selection theory attributes men's 'taste for risk' to a form of mating display [29]. Second, a widely held explanation is that men may lack the knowledge to prevent and detect health problems which influences help seeking [13,20,27]. And third, men may actually be aware of health issues and of their bodily experiences, but feel uncomfortable when they feel vulnerable. Illness may threaten masculine ideals of dominance and self-reliance [25] and men may delay help seeking because first they want to be sure they cannot 'fix' it themselves [28]. These explanations all suggest that beliefs about masculinity play a role and are one predictor of risk behaviour [13]. Men's health risks such as substance abuse increase with stronger endorsement of norms of masculinity [13,21,30].

### Gender theory and hegemonic masculinity

The concept of hegemonic masculinity refers to a pattern of practice that allows the domination of men over women and which is also to be distinguished from other masculinities [6]. Hegemonic masculinity is defined as the most widely accepted and normative form of being a man in a society, which is to be placed within a gender order that assumes men and women are mutually exclusive categories [6,31,32]. Hence, to be a man is to be not-female and vice versa, but the concept of masculinities goes beyond dichotomous sex differences and refers to the diversity and complexity between men and forms of masculine identity across situations [6,32,33]. Gender is, therefore, best understood as a dynamic, social structure in which power relations between men and women as well as between men and between women are important, although no grand structure of power exists in which some men have power while others - men or women - have none [6,7,13,24,34]. The active participation of men in the construction of their masculinity and masculine behaviour is considered 'doing gender' [13,35]. Besides hegemonic masculinity, Connell also described other masculinities, such as complicit masculinity, which refers to men who neither challenge nor conform to hegemonic masculinity but rather benefit from those men who demonstrate hegemonic masculinity. Subordinate or even marginalized masculinity may be more prevalent among homosexuals, ethnically different masculinities, or men suffering from illnesses or disabilities. Other background identities such as sexual orientation, age, class, health or ethnic background intersect with gender [1,6,36]. Among other things, studies of masculinity aim to understand how certain masculinities are related to risk-taking behavior and ill-health [13,17,25]. The pursuit of hegemonic masculinity, which incorporates a concern with success and dominance, may mean physical strength for young men while for older men career success may play a larger role. Besides, some masculine ideals may be more important in certain contexts whereas they lose strength in other contexts. Health-related beliefs and behaviors may be ways to construct masculinity in a given context and the pursuit of masculinity may also imply healthy behaviours [13]. For instance, Williams found that boys with asthma or diabetes felt more in control of their chronic condition than girls [8]. The boys made every effort to keep the chronic illnesses out of their personal and social identities by taking their medication as invisibly as possible and by stressing the importance of exercise. However, some boys could not 'pass' as healthy when their symptoms could not be ignored. Consequently, this led to disparaged masculine identities [8]. Equating masculinity with success may make it more difficult for men to accept being ill [31], even though men may also aim to respond to their health needs in a timely manner [28]. Men may dislike seeking help too early and the capacity to maintain regular activities and every day tasks influences help seeking [28]. Aging men mention control over chronic disease as a way to maintain independence and to maintain daily functions [37].

### Male employees, masculinity, and workplace physical activity

Employers are traditionally involved in occupational health safety issues; legislation and policies force them to prevent injury and occupational disease and safeguard healthy working conditions. In the past decades in the Netherlands, large progress has been made. Furthermore, changes in the social insurance scheme delegate financial responsibility for sickness absence leave and work disability to employers. Therefore, workplace health programs focus more often on preventive measures and health promotion. Health becomes a larger concern for employers. They want to reduce health and financial risks and improve economic and productivity aspects [38,39].

Recently to target men's health issues, Australian and Irish governments developed men's health policies including worksite health promotion [20,39]. For instance, establishing supportive working environments such as age-specific health checks and services in the workplace aim to empower men to prevent health problems and seek help. Tenets of such policies should be that different health promotion approaches appeal to different men under different conditions [40].

Generally, worksite health promotion programs include a workplace physical activity program, provide advice and information, or do both. Factors known to influence participation in worksite health promotion programs are: unequal access for employee subgroups across age and education levels [41]; perceived health, the intention to change risk factors, psychological well-being, and job pressure [42]; difficulties in reconciling exercise with work and care responsibilities, lacking support at management level, and personalized services and high-quality supervision [15].

Further understanding of factors that encourage or discourage men's participation in lifestyle interventions such as workplace physical activity is necessary. In general, sports are a context where people can enjoy competitive play and gyms are places where people work on vitality and looks in order to be successful in society [10]. The rising popularity of aerobics/workout is heavily informed by beauty norms to control body shapes. Initially, women propelled the increase of aerobics to control their weight, while later men's interest in bodybuilding has stimulated muscle building workout [10].

### Aim of the study

Men and women differ in their health behaviours, including risk-taking and seeking help [1,13]. In regards to working out, gender-specific preferences exist in the particular activities men and women carry out in gyms. For men, sports may be an important source of emotional attachment, belonging, and identity either as a player or as a spectator [32]. In addition, concepts of masculinity are largely defined and reinforced within the realm of work [31], which is a primary source of identity for men [13,25]. Robertson developed a framework for the relationship between health and hegemonic masculinity and argues that men must resolve two conflicting discourses: first, 'real men' do not care about health; and second, good citizens are morally required to pursue health. This tension is called the 'don't care/should care' dichotomy in which men must find a balance. Furthermore, adhering to disciplinary health regimes on the one hand (control) must be balanced by pleasure (release) on the other hand [43]. This study is to be placed within the concept of social constructivism because it discusses and questions of how masculinity is constructed in relation to health beliefs and vice versa [13,25]. Little empirical evidence from the Netherlands exists about how masculinity ideals are enacted by individual men in specific health contexts. In order to adapt workplace physical activity to men's health needs, more insight is required into how male employees perceive health and health promotion in general and workplace physical activity in particular. Such perceptions might be related to their pursuit of masculine ideals, which then have consequences for men's participation in workplace physical activity. Therefore, we explore men's health beliefs and attitudes towards health promotion, in particular workplace physical activity.

## Methods

A qualitative individual interviewing approach was chosen to gain a deep understanding of the men's lives, health beliefs and behaviours, and perceptions of masculinity. The interviewer was a young, heterosexual, Dutch white male graduate student in his early twenties who had an interest in masculinity and health and had recognized that, recently in his life, masculinity norms had heavily influenced his health behaviours. Hence, the interviewer's identity (HS) as well as his experiences informed the study objectives and background and influenced the interview setting. Nevertheless, we contend that the interviewer's expressions and the interviewees' talk have a generality outside the interview context. The way the men talk does represent 'the social within the psychological' [33]. The interviews were based on an interview outline (see Table 1) and lasted approximately one to one-and-a-half hours.

**Table 1:** Interview outline

Interviewee characteristics	Name/age/marital status/children/ethnicity Sexual orientation *(not brought up straightforwardly; discuss at the right time. Depends upon the interviewee and may thus be discussed by the end of the interview. Interviewees are not pushed to answer)* Participation in workplace physical activity?
	
Company	Core business of the company How large is the organization Health and lifestyle interventions offered?
	
Can you describe your job?	Responsibilities/tasks
	
How would you describe your health?	Ideal health situation Health beliefs and behaviour Comparing eating habits to your ideal What is healthy exercising to you and how often should that be? Exercise and how often Smoke and how much Do you experience disadvantages of smoking Desire to quit/Reasons to quit Leisure/hobbies Paying attention to health during leisure time, why (not)?
	
Often excessive drinking is defined as drinking 6 glasses or more by occasion.	What do you think about this definition? Does it apply to you?
	
Introduction about typical examples of masculinity in every day live. Examples about work, friends and sports like soccer.	What is your image of the ideal man? What do you consider masculine and what not? What, according to you, are positive and negative aspects of masculine behaviour?
	
When looking at your everyday life, does masculinity play a role in your:	Home situation Circle of friends Work situation Health beliefs and behaviour like eating habits, exercise, smoking, drinking and leisure time
	
What do you think about workplace physical activity?	Own possibilities for workplace physical activity Why does employer offer it Which factors determine (non-)participation

In line with recommendations about interviewing men [44], an interview outline was developed, used in a pilot-interview and was accommodated according to feedback from the interviewee and the experiences of the interviewer [44]. The phrasing of questions, the structure of the interview, as well as the experience of discussing these issues among two men was discussed directly after the pilot-interview and afterwards among the researchers. Men prefer to be introduced by mutual friends that may encourage them to participate in interview studies [44]. Providing specific research questions before the interview allows the participants time to process thoughts and opinions and reflect on the issues at hand [44]. Therefore, we informed the men beforehand by email about the issues that would be discussed. Another important issue is that men deduce the interviewer's orientations such as his educational background (in this case, health scientist) before and during the interview and hence, they develop their answers in a gendered context. The men may try to avoid the impression that they are not masculine [44], or may find it difficult to talk about health because commonly they do not discuss it [45]. Sharing experiences for instance about hobbies may help to build rapport [28]. Although this practice may reinforce stereotypical masculine exchange in the interviews, it may also create the proper environment to discuss more intimate topics and challenge masculine aspects [28,46]. Robertson [47] explores how being a male researcher investigating gender played a role in interview dynamics with men. In our study in four interviews, the interviewer had difficulty with openly asking about the men's sexual orientation as a direct response to the homophobia he experienced. Three of these four men were married to or cohabited with a woman. In the other interviews, the question was directly posed. The interviewer's experiences confirm observations of the do's and don'ts in interviewing men about health [44] but also provide evidence for our finding that masculinity is related to not disclosing vulnerability. By not asking about their sexual orientation, the interviewer felt insecure and avoided challenging the interviewees' masculinities. This mirrors Robertson's unease when relating to gay men, because he does not want to be misrepresented as a gay man himself. He calls this "another example of the deeply embedded nature of homophobia within the construction of hegemonic male identity" [[47], p. 314]. Other concerns during interviews with men are that some initially try to take control over the interview [44,46], which may provide information about how closely the men align with expressing masculinity through dominance [44]. Other men may find it difficult to talk and few men tolerate long interviews. Interviewing skills and alertness for body language that transfers participant fatigue is important [44]. Thirteen men aged 23-56 years who were offered workplace physical activity were interviewed once in the fall of 2008. Their names are aliases to maintain confidentiality (Table 2).

**Table 2:** Overview of participants

Names	Age	Civil status	Children	Work-place physical activity	Sector	Job	Sexual orientation
**Jerry**	31	Coha-biting	0	Yes	Mental health	Clerk	Unknown
**Ben**	54	Married	2	No	Mental health	Technical service	Unknown
**Woud**	28	Single	0	No	Advertisement	Agent	Unknown
**Jan**	53	Married	3	Yes	Mental health	Therapist	Heterosexual
**Maarten**	39	Coha-biting	0	Yes	Mental health	Clerk	Heterosexual
**Tom**	47	Divorced	2	No	Transport	Truck driver	Heterosexual
**Roan**	42	Married	2	No	Posting	Commercial employee	Unknown
**Edwin**	34	Coha-biting	0	Yes	Mail order business	Social worker	Heterosexual
**Berend**	40	Married	2	No	Media	Manager	Heterosexual
**Martijn**	23	Single	0	No	Transport	Courier	Heterosexual
**Raymond**	29	Single	0	No	Transport	Truck driver	Heterosexual
**Rob**	35	Coha-biting	0	Yes	Advertisement	Office assistent	Heterosexual
**Bart**	56	Married	2	Yes	Oil industry	Team leader	Heterosexual

The men were of different ages and they work in a wide range of professions, at different education levels, and in different sectors. All were offered workplace physical activity by their employer, but we explicitly invited both exercisers (6) and non-exercisers (7). Men were enlisted through a snowballing method via personal contacts of the interviewer. These personal contacts introduced us to colleagues or to other contacts. The interviewer and interviewee did not know each other. We used this method to find a purposive sample of men to ensure that a range of men were represented. Such variation seeks to maximize the breadth, depth and richness of the data, which increases the validity of our study. Various issues raised in earlier interviews could be explored in more depth in the following interviews. All interviews took place in the men's workplaces or homes. Interviews were tape-recorded, transcribed verbatim, and open-coded. Data analysis was conducted by means of a thematic analysis method to examine underlying ideas and assumptions that shape or inform what is said in the interviews [48]. Two of the researchers (HS, PV) reached consensus in regards to coding, codes were clustered into categories which have been thoroughly discussed, after which key themes were identified (researcher triangulation) [48]. The validity of the study was further enhanced by analyzing memos based on observations during the interviews and after transcription was completed (method triangulation). After theoretical saturation was reached, when no new issues and concepts came up in the interviews, no further interviews were held. All three authors connected the themes to the theory. In the Netherlands, ethical approval for an interview or survey study with employees is not required (see for Dutch legislation http://www.ccmo-online.nl/main.asp?pid=1&taal=1).

## Results

Two main normative categories emerged from the data: (1) *The ideal man is a winner: Masculinity and competitiveness; *and (2) *A real man is not a whiner: Masculinity and health issues*. Being two sides of a coin, these two normative categories somehow represent upper and lower limits of how men should be, and encourage and discourage participation in health in different ways.

### The ideal man is a winner: Masculinity and competitiveness

An important way mentioned by the interviewees to try to achieve or expose masculinity is by exposing their performances to other men. In the interviews, high achievements in sports, alcohol use, or other physically challenging risk taking behaviours are mentioned. It is vital that these achievements are noticed by other men or their value diminishes. The importance of dominance and public performances can also be derived from their image of the ideal man, which surprisingly does not differ much among the interviewees. According to most interviewees, the ideal man is successful in multiple areas such as sports, work, and risk taking behaviour but also as a father and a husband. To shortly summarize, the ideal man is a winner everywhere and always. Despite the ideal, all men recognize that being a winner always is not feasible and hence, the 'real life' standard is to at least try and be one. In order to show masculinity and gain respect, men are required to compete. Almost all men associate masculinity with impressing other men in the workplace and in regards to career and sports. Despite the possible variation in masculinity, a clear border of masculinity is sexual orientation; being gay is not considered masculine. Avoiding any association with femininity is important because other men will lose respect. For instance, several sports are mentioned as women's sports: ballet, Nordic walking, horseback riding, or netball. Rob explains that he would not play netball, because it is a women's sport and therefore, an embarrassment:

I: But would it hurt your masculinity if other men knew you played netball?

*"Well it isn't that bad but a little, yeah. You are not really a tough guy that other men look up to and think: look at him, he plays netball. They would rather laugh at you than show respect for you." *(Rob, 35 years)

When men are involved in a feminized sport, they have to be particularly good and passionate about it to gain respect from other men. Competitiveness is also shown by trumping each other by humour. Drinking a lot is often considered a high achievement and plays a role in establishing a position among men. Large networks with many friends are important and male friendships contribute to men's well-being. Raymond has to prove his 'equality' and therefore it seems that competition is part of the 'fun' experience among friends to which alcohol contributes.

*"I just want to prove that I am nothing less than my friends. When we are together we generally drink some beers, I am certainly not going to drink cola when we are having fun." *(Raymond, 29 years)

The interviewees state how men try to impress each other in work, sports and in other situations such as discussions, risky behaviour, using humour and joking, and story-telling. Some men also recognize a downside to trying to impress others, for instance in friendships:

*"I had to book a short holiday with friends, something we do every year. But I was so determined to exceed the previous holidays in the years before that I had booked a hotel that was much too expensive and luxurious. I totally forgot what the essence of this holiday was. I was only busy making others look up to my perfectly organized holiday." *(Maarten, 39 years)

In other domains, such as competitive sports, winning is a more clear-cut goal. And although workplace physical activity is not a competitive sport in itself, Jerry likes to show off in the gym:

*"I like to show my colleagues that I am athletic. There is a competitive atmosphere between me and my colleagues. Maybe it is a way to demonstrate my capabilities, I think proving myself plays a role in this." *(Jerry, 31 years)

Besides liking to prove themselves, the men are also often *asked *to prove themselves:

*"Well, I am often challenged by my colleagues. They frequently say, come on old man, show us what you got. And then I have to accept that challenge. I cannot let it pass because I still want to win." *(Jan, 53 years)

Rejecting the challenge already detracts from their masculinity and, thus, affects their position in male hierarchy. Nevertheless, some men perceive the requirement as a burden. Feelings of pressure may exclude men from certain contexts; they want to escape knowing they will not live up to expectations, or they may feel they prove their masculinity in other contexts. Age may play an excluding role in workplace physical activity. Jan, an older exerciser, said in the excerpt how he 'still' wants to win which suggests that he expects eventually an ending to this desire. Ben refers to feeling old being around young participants, and Tom thinks workplace physical activity is something for younger men:

"Only young guys participate in workplace physical activity. I cannot see myself standing between those young guys. Besides, I think after ten minutes I am too tired to continue. I just don't want to embarrass myself."

I: Are you ashamed to participate in workplace physical activity?

*"Well, being ashamed is a bit of an exaggeration. But, I am not very athletic and it is hard for me to just step in and participate. Besides, I do not even know how these fitness machines work. No, I think working out is something for young men." *(Tom, 47 years)

Comparison with other men is important. The men expect having to compete with athletic, possibly younger, men in the gym - not being athletic would be an embarrassment. The interviewees do not expect women to be around or at least they do not refer to them. Other younger men distance themselves from exercisers by framing masculinity differently. For instance Woud, a non-exerciser, challenges the muscle building norms he expects in workplace physical activity. He does not moralize that people should exercise for their health, but when they exercise, fun and health should be their motivation and men should not exaggerate looks. Martijn opposes the type of masculinity that is, according to him, displayed by male participants of workplace physical activity:

*"They are only occupied with their looks; they are not fun to hang out with. You cannot really laugh with them and I always get the feeling I have to prove myself. Besides, when there is trouble such as a stressful situation at work or when they have a cold they are the first to complain about it. Furthermore, they may look strong with those big, muscular arms but they are not. It is like they have air in their arms." *(Martijn, 23 years)

Again, fun is mentioned, and according to Martijn the male exercisers are not fun to be around. He feels he has to prove himself to the exercisers who seem physically stronger than they really are but whine about a common cold. According to him, real masculinity is exposed in having a good sense of humour, handling work stress, or stoicism in times of illness; they may think they are winners, but according to Martijn, they are not.

The existence of hegemonic masculinity is obvious to the men, although for different men different aspects are most important, and some hegemonic masculinities are resisted or not complied with. 'Real' masculinity means aiming for, but not necessarily reaching, the 'ideal'. Competitiveness is required to show masculinity to other men. Older men in particular, but also some younger men seem to refrain from workplace physical activity because they resist the type of masculinity, characterized as young, obsessed with muscle building, looks, being physically strong, and dominant in the gym. Furthermore, the men relate workplace physical activity to fitness equipment and do neither consider other physical activities nor do they mention the participation of women.

### A real man is not a whiner: Masculinity and health issues

In regards health issues, hegemonic masculinity seems strongly expressed in male attitudes towards (in)vulnerability. According to most of the interviewees, real men should not be taking care of their health when there is no need; this would expose weakness and feelings of vulnerability. Most of the interviewed men relate invulnerability positively with being masculine and dominant and they desire to perceive themselves as invulnerable. This forms an obstacle for self-care only until psychological or physical health complaints can no longer be ignored. Most of the men consider themselves healthy because they do not experience health problems even if they would, for instance, smoke or drink too much. According to the interviewees, men only talk about health when there is something wrong, and there should not be something wrong with it. This gives the impression that men find their health uninteresting, and this indifference or nonchalance then becomes another source of masculine pride. In this theme, the physical results of working out are not necessarily associated with being competitive but rather with self-confidence and nonchalance ("looking fit").

Among men, only health problems that are large, serious, and solvable and affect work can be safely discussed. Using humour and joking is an outlet to discuss emotions or problems without being too serious about it and without coming across as vulnerable:

*"It is just an easy way to discuss certain problems. When you tell them in a funny way you can place the problem in perspective. And it makes it light instead of dramatic." *(Roan, 42 years)

Outsourcing self-care, hence outsourcing femininity, to female partners allows some men to take care of their health without harming their masculinity ideals. The role of women in taking care of men's health is vividly described by Roan, who mentions his difficulties with seeking help when he is ill:

*"After some time of illness, my wife starts pushing me to go to the doctor. And the difficult thing is, that most of the time she was right that going to the doctor was necessary. But, you do not go to the doctor for each little thing." *(Roan, 42 yrs)

The role of his partner liberates Roan from masculine norms to not take care of his health and disclose his vulnerability while maintaining his sense of masculinity: he goes because she thinks it's important. Not disclosing vulnerability also plays a role in relation to the men's family. On the one hand, it is to be placed in their sense of duty to protect the family, because a man should provide security and be a role model:

*"I think a man has to be strong and should not cry over every disappointment in his life. Besides, he has to set an example for his kids and crying would not really help him. As a father, you like to show your kids how to be a real man. And set a role model of what a good father is like." *(Ben, 54 years)

On the other hand, being a father legitimizes that men feel and show vulnerability. Some men with family responsibilities are allowed to pay attention to their health without it negatively affecting their masculinity or position within male hierarchies. Being a role model motivates Roan to admit to his daughters that he should have taken better care of his health:

*"I have two daughters; they are twins and 16 years old. You are right I do not set a good example by smoking and I should stop for them. But, I do try to warn them for the negative effects of smoking and I hope they will never start smoking. And I also tell them honestly that if I could turn back time, I wish I had never started smoking." *(Roan, 42 years)

Complaining or being emotional about minor problems is something that women would do and, hence, something to avoid. But sometimes, the men feel vulnerable and they need to discuss what they call 'vague' or emotional problems. Often, such problems can be safely discussed with women:

*"Showing emotions is easier with women. I think women are better capable to understand your emotions when you are struggling with something that is not a very clear problem. It is just nicer to talk about problems with women than with men, I do not really know the reason for that." *(Martijn, 23 years)

Tom, who said that his focus on work earlier in life had contributed to his divorce, even goes as far as calling his *ex-*wife when he has emotional problems, but other issues can be safely discussed with other men:

*"There must be problems men can help each other with; otherwise, you should not mention them. But when I am having problems I call my ex-wife. I can tell her everything and it is a lot easier to talk about *[such problems] *with women." *(Tom, 47 years)

Tom presents as a traditional family man who only began to take care of his health after his physician had said so:

I: Do you think it is typical for men to not pay attention [to health] too much and only take action when someone like a physician says something?

"Well, as a man you, you will not pay attention to your health daily, no. That is more something for women. Besides, I had never had any problem before."

I: Okay, but why isn't that something men pay attention to?

*"Well, I just wasn't very interested. And I do not start whining about every little complaint. As a man you just work and provide for the family and pay for expenses." *(Tom, 47 years)

Tom's remark exemplifies how men may perceive their health in a more pragmatic way: health is not interesting unless daily functioning is impaired. In the excerpt, the interviewer responds to Tom's presentation of a man who complies with conventional hegemonic masculinity. However on Saturdays, Tom does household chores, because there is no *"wife anymore who does these things for you*." He does not seem to have problems with doing this work although he states that men are providers for the family who do not complain about little problems, and do not discuss intimacies with other men. By the end of the interview, Tom asks:

"Ok, but don't you find it weird to speak with men about these issues? I mean, I do not mind you know, but sometimes they are rather intimate issues."

I: Yes, I have to get used to it. But it goes a little better with every interview. I find it in particular hard to ask about how people feel or how they experience things.

And then Tom reassures the interviewer:

*"I can imagine that. But I actually thought it was a nice conversation, you know." *(Tom, 47 years)

This quotation shows that openly admitting to vulnerability may be difficult for men. Nevertheless, Tom directly asks the interviewer how he experiences discussing intimate issues, which is rather intimate in itself. This interview context, with a younger and uncomfortable male interviewer, allows Tom to make this statement without damaging his masculine identity.

When asked about health, almost all men relate health to sports or exercise but sports or exercise is hardly related to health. Competition, fun, and risk-taking seem more important than health, only when sports are fun, can they be healthy:

*"I think it is healthy when people do sports because it gives them a good feeling and they like it and do not sport because of the 'healthy'." *(Woud, 28 years).

Alcohol abuse is often referred to in the interviews as a way of showing masculinity by competing and sharing friendship but also a means of escaping masculinity and losing control. Openly showing healthy lifestyle behaviours is not masculine, while openly showing unhealthy behaviour is considered masculine. Interestingly, men who do not adhere to that norm such as Maarten, who puts effort into his health, do not contest it either but refer to the other domains where they have the highest achievements:

*"There is a certain hierarchy within that atmosphere. They are the boozers; they are on top of the hierarchy when looking at the group when we are in the pub. I am on top of the hierarchy when it comes to sports." *(Maarten, 39 years)

For Maarten, being masculine in sports outweighs the fact that he is not masculine in drinking but the reverse is also true for other men. The men seem to constantly balance healthy and unhealthy behaviours and these balancing acts determine the health price they are willing to pay for being masculine:

*"The perfect balance is when someone lives a healthy life without missing the fun aspects of it." *(Rob, 35 years)

*"I think you should enjoy life, and drinking alcohol or eating unhealthy food from time to time is part of that." *(Roan, 42 years).

Balancing short and long term health goals sounds fairly reasonable. However, the possibility that short term goals (fun with the guys in the pub) must be constrained at least a bit in order to reach long term health goals is hardly considered. What men consider 'from time to time' does not always concur with established norms. When confronted with the official alcohol health limit of five consumptions per occasion and a maximum of 21 consumptions per week for men, many of the interviewed men - including those who hardly drink - challenged its legitimacy:

*"Well, if that is the case I seriously have to think about it because I drink more than 6. But I do think it is nonsense. I mean everything that gives pleasure is considered as bad." *(Tom, 47 years)

From the interviews, other health knowledge appears to be present. Another matter is showing openly that you possess that knowledge or that you live a healthy life for your *health*. Jerry does associate sports and exercise with health, but for him this also seems connected to 'feeling good' and to looks:

*"I did sport fanatically from an early age on. I just feel good about it and health is my top priority. And a part of it is also vanity I think *(laughs)."(Jerry, 31 years)

And Maarten really takes care of himself in terms of healthy nutrition, self-care, and exercise, but health only vaguely motivates his behaviours:

*"I like to look good, not just for myself but also for my girlfriend. For me, someone is masculine when he looks good and fit. Furthermore, he has to show toughness and nonchalance. When you look good, you feel good. It gives me a feeling of safety and self-confidence." *(Maarten, 39 years)

Earlier, other men had recognized that workplace physical activity is for men who find looks important and want to come across physically strong. The quote above put 'looks' in a more psychological perspective. By looking good, showing toughness and nonchalance, Maarten tunes into a self-confident masculinity that protects him from feeling insecure and vulnerable. Looking good, presented in alignment with being fit and masculine, is mentioned by the exercisers. Health behaviours, preventive behaviours, or help-seeking seem not very masculine. But some men can legitimately pay attention to their health when they are role models for their children or when they actually have clear-cut health complaints. Workplace physical activity may help men to construct a certain type of masculinity, expose vitality, and be competitive. Looking good is important; sports or exercise may contribute to looking 'fit' and self-confident. Workplace physical activity, however, does not tune into ideas of what a good worker should do or is like, and it is hardly mentioned as an instrument to improve health.

## Discussion

### Main findings

We distinguished two themes in our study among 13 white Dutch male employees who were offered workplace physical activity programs. The first theme *'The ideal man is a winner' *encompasses the perception that, ideally, men are winners. In reality, men recognize that this is not feasible, but at least it is masculine to be competitive and noticed by other men. This fits other authors' work on hegemonic masculinities and the existence of hierarchies among men [6,27-29,49]. The interviewees perceive workplace physical activity in relation to looks: either to pursue a particular type of masculinity (young, occupied with looks, interested in muscle building) to compete with if they participate or to look 'fit'. Other than muscle building activities such as stretching also offered in gyms are not mentioned. In line with this, Robertson showed that men in the context of cardiac rehabilitation preferred a 'vibrant physicality' after exercising over a 'relaxed physicality' after yoga [43,50].

A second theme is *'A real man is not a whiner*.' Although the ideal is to be invulnerable, the men are quite decided about 'real men' not *feeling *or *disclosing *vulnerability; they do not complain about minor health issues. Health messages for instance about alcohol intake are openly rejected and taking care of health is conceptualized feminine, hence, something to avoid. Our findings that many men are careful about disclosing vulnerability, and rather outsource self-care and the decision to seek help to female partners are in line with other studies [13,26,27,45]. This allows the men to take care of their health in ways that do not harm their masculinity ideals. Looking 'good' or 'fit' as a result from workplace physical activity is associated with not feeling vulnerable. In line with this, Robertson reported that some men associated obtaining or maintaining 'fitness' with resilience or resistance against chronic disease [50]. In our study, workplace physical activity is hardly framed as a health measure and it is not associated with their worker role or their productivity.

The two themes 'being a winner/not a whiner' seem to be two sides of a coin to which other men are the social context by providing peer pressure. Men do compete over coming across invulnerable and self-confident, and they should at least not whine when facing setbacks.

Our study supports theories that gender is a way in which social practices are ordered and that such gender constructions are important for health beliefs and possibly behaviours. Cultural notions of the relationship between masculinity and men's health beliefs are relatively stable [51]. In line with Connell's theory on hegemonic masculinity and with earlier literature [6,17,27,31], our findings suggest that hegemonic masculinity ideals may come at the expense of men's health, although not unequivocally. Studies suggest for instance that both low as well as high masculinity scores are related to adverse health outcomes such as increased risk for CHD [52,53]. The equation of health measures with femininity places masculine ideals in opposition to positive health beliefs [13,17]. Our study also confirms theories and findings that men's health beliefs vary with the type of masculinity they pursue and across contexts [6,13,17,25]. The "don't care/should care" model provides a good understanding for our findings [43]. This model predicts ambivalence, because it causes men to live up to or comply with hegemonic ideals in some contexts while rejecting them in other contexts [33,54]. Furthermore, the model predicts that disciplinary health regimes (control) must be balanced by pleasure (release) [43]. For some interviewees, adherence to health regimes was masculine when it allowed them to compete with other men or to feel self-confident. Others claimed that unhealthy behaviours, framed as enjoying life or presenting as invulnerable, were masculine. We did not find that the Dutch men in our sample articulated a concern with health as a moral requirement or that they 'should care.' We rather found 'should have cared' as a concern, in order to be a role model for children. Thus, the single role particularly affecting concern with health was being a father and thus a role model. The work role or other roles were hardly related to concern with health.

In our study, masculinity beliefs encompass the notions that men should always be prepared to compete and avoid disclosing vulnerability because that is feminine. Nevertheless some men do household chores, pay attention to nutrition; disclose intimacies, or regret smoking. Hence indeed, masculine ideals do not correspond tightly to the lives of actual men, but are loosely in line with how the men constitute masculinities as ways of living in everyday circumstances [54]. Likewise, framing health promotion where following health advice is a moral requirement is also resisted by many men [23,40,43].

The finding that health is women's, not men's, business and responsibility and that men keep quiet about their health problems is reported, for instance, in a study on prostate ill-health [55] or on help seeking among men [13,27], although others found that men openly discuss health issues in the proper environment [37].

We did not replicate the finding that men know little about health. This does not mean that men and women possess equal knowledge of their bodies in all health aspects. For instance, regarding reproductive health issues, men may be less aware of their bodies than women [55]. Our interviewees seemed quite aware of what was considered healthy or not. Often, they rejected such considerations, which is called reactance; a negative reaction to messages that are perceived to reduce autonomy [23]. Regarding sports, Dutch men are as aware as women of its importance for health [56] although, in our study, workplace physical activity was not framed as a health measure but rather as a way to control body shape.

The fact that the interviewer was young and male offered a different social context than when a woman would have been the interviewer. As gender researchers, it is impossible to refrain from the gender system as objective outsiders [25]. Most men were clear in their statements that discussing emotional issues with women was easier which may have played a role in the outcomes. This may stereotype women's role in conversations as the unthreatening empathic listener, but such rather benevolent 'sexism' may also have a hostile side depending on the conversation topic and the environment [57,58]. Thus, Tom's question to the interviewer about discussing intimate issues with men might or might not have been asked to a woman. Power relations are created within interview settings and therefore interviewers must be aware of dominant perspectives stemming from social locations such as maleness, whiteness, or education [45,59], but also from the interviewer's age and self-confidence [58], as our analysis confirms. Such reflexivity can lead to another form of understanding of the interviewee. To study masculinity ideals, a male interviewer had clear advantages. The interviewer himself and the topic may have primed some men to talk about health differently than when masculinity had been excluded.

### Limitations to the study

We have interviewed men at different ages and education levels and in different occupations; despite variation in health beliefs and differences in compliance with masculinity norms, participants held similar beliefs about the ideal man. This is in line with the statement that the concept of hegemonic masculinity regulates men's conduct by shared norms, although men can be both compliant as well as resistant at the same time [33]. Nevertheless, our sample is rather small and we have interviewed heterosexual male employees with Dutch ethnic backgrounds. In the Netherlands, subordinate or even marginalized masculinities might present ethnically different masculinities, homosexual men, or men suffering from illnesses or disabilities. Other background identities intersect with gender to present different forms of masculinities [1,6,43]. Life events such as unemployment or disease may also trigger a redefinition of masculinity and change men's health beliefs [27,28]. This may influence whether the men aim for, reach or maintain hegemonic ideals, or whether they reach a 'no man's land' as defined by Robertson [[43], p. 185]. For some men, room may emerge to take up responsibility for their health and re-imagine their masculine identity. In line with other studies [27,37], our study suggests that when health complaints can no longer be ignored and can be perceived as problems that need resolve, some men do pay attention to their health. Furthermore, the physical body that is 'fit for work' may play a different role for working-class men than for academic men and engagement in work among men differs in the face of illness [60].

Our focus on lifestyle behaviours may have downplayed other factors affecting men's health. In this study, we did confirm masculinity as rather negative towards health beliefs. Men are often blamed for being their own enemies, but maintaining a job, bringing home earnings, and maintaining friendships are also important to health and well-being [28,37,40]. This points towards a more pragmatic embodiment which means being in a shape that allows men to fulfil everyday roles and tasks [28,40]. Besides, there is no direct connection between health beliefs and actual behaviours [51]. Men may take care of their health in many ways.

### Implications of the study

'The hegemonic masculinity of workplace physical activity' is represented by a young male concerned with body shape, in particular muscle building, who sends out a competitive message to other men. Our study indicates that some (for instance aging) men may withdraw from sports and workplace physical activity when they cannot live up to or resist certain masculine expectations. This is rather disturbing from a health promotion perspective. Workplace physical activity seems narrowly defined and the perceptions are in line with how aerobics/workplace physical activity historically developed [10]. Marketing workplace physical activity as a wider range of activities than fitness machines and muscle building may contribute to higher participation rates among men. Generally, men find competitive activities more interesting than women [11] and we conclude from our study that the men introduce competition in workplace physical activity while it does not aim to be competitive. Men may be put off when a focus on health or on body shape is too obvious. Promoting health in the workplace seems particularly acceptable for men [20,43]. They may indeed be reached in the workplace, but more hurdles must be taken.

A life course approach to studying masculinity and health beliefs and behaviours may shed light on their development, for turning points in men's lives may affect masculinity ideals, their health, and well-being [27,37,40,50,51]. In a quantitative study, in particular men find that the necessity for high achievements in sports is overrated. Young people aged 15-19 years old and those older than 50 find competitiveness in sports more important [56]. Younger men may enjoy competitive sports more often; becoming a father may positively contribute to men's self-care; while older men may be more inclined to seek health care. This stresses again the need for a multi-faceted approach to health promotion to engage different groups of men in different ways and in different settings [20,40]. Future studies may focus on how men's pursuit of masculinity and rejection of femininity influence public health interventions at different levels, such as reach, implementation, or maintenance of health behaviours [41,61].

In order to increase male participation, two potentially conflicting types of interventions may be developed. The first type is grounded in the perception that masculinity beliefs are here to stay. Department competitions or monitoring individual improvement may be ways to attract and keep one group in the program, while relieving other men in individual programs from having to compete is also important. This carries however the risk of reproducing masculinity as an explanation to excuse for men's beliefs and behaviours [54] and maintains the assumptions that 'boys will be boys'. Men's health beliefs can be targeted by leaving masculinity beliefs intact or even strengthening them [40]. Such interventions are not uncommon; the aggressive marketing of sildenafil (Viagra) and its widespread use is a clear example. Other messages within this line of thinking may focus at how masculinities deteriorate as a result of unhealthy behaviours. Health promotion messages may for instance explain how alcohol disinhibits sexual arousal, but inhibits sexual responding [62], or point towards the relationship between smoking and erectile disorders [63]. A second type of interventions aims towards challenging masculinity beliefs. This is also important to establish gender equality and shift the gender order to benefit both men and women [9]. Our findings suggest that it is not necessarily doing certain types of masculinity, but in particular in combination with avoiding femininity that puts men at risk. This feeds the notion that gender indeed is a relational concept [6,32,51]. According to Sabo and Gordon [30], more men ought to refuse to be *men*. Men need support to take care of their health, include healthy beliefs into notions of self-reliance, learn how to appraise certain situations and activities as non-threatening to their masculinity [21,13], model their behaviour more to what is stereotypically called 'feminine' [13,64], and find ways to transform risk-taking into less health damaging and yet challenging adventures. Researchers advocate empowerment initiatives to target gender stereotypes for both sexes [5]; but a focus on empowerment of men in health promotion, for instance in sex education, is not always in the interest of social justice and gender equality [65]. Interventions and policies must therefore explicitly relax the constraints of rigidly defined gender roles [64,65].

## Conclusions

Two normative themes were found: first, ideal men are equated with being winners and real men are prepared to compete, and second, real men are not whiners and ideally, not vulnerable. These themes are related to men's health beliefs and influence their perception of workplace physical activity. Some men resist what they perceive to be an emphasis on muscled looks, whereas for others it contributes to looking self-confident. Up to this date in the Netherlands, the social and economic gains of masculinity outweigh the losses, including health loss [64]. Taking the gendered backgrounds of lifestyle behaviours into account (for instance aging men's resistance to ideals of the muscular masculine body) in worksite health programs including workplace physical activity is important. Such interventions will improve by greater insight in the tensions between health behaviours and beliefs about masculinity and a focus on changing these beliefs.

## Competing interests

The authors declare that they have no competing interests.

## Authors' contributions

HS conducted the interviews. PV and HS reached consensus as regards coding and all three authors connected the themes to the theory. PV and HS conceived of the study and participated in its design and coordination. All authors helped to draft the manuscript and read and approved the final manuscript.

## Pre-publication history

The pre-publication history for this paper can be accessed here:

http://www.biomedcentral.com/1471-2458/10/712/prepub
